# PPAR-γ Ligands Repress TGFβ-Induced Myofibroblast Differentiation by Targeting the PI3K/Akt Pathway: Implications for Therapy of Fibrosis

**DOI:** 10.1371/journal.pone.0015909

**Published:** 2011-01-06

**Authors:** Ajit A. Kulkarni, Thomas H. Thatcher, Keith C. Olsen, Sanjay B. Maggirwar, Richard P. Phipps, Patricia J. Sime

**Affiliations:** 1 The Division of Pulmonary and Critical Care Medicine, Department of Medicine, University of Rochester School of Medicine and Dentistry, Rochester, New York, United States of America; 2 Department of Microbiology and Immunology, University of Rochester School of Medicine and Dentistry, Rochester, New York, United States of America; 3 Department of Environmental Medicine, University of Rochester School of Medicine and Dentistry, Rochester, New York, United States of America; 4 Lung Biology and Disease Program, University of Rochester School of Medicine and Dentistry, Rochester, New York, United States of America; Cairo University, Egypt

## Abstract

Transforming growth factor beta (TGFβ) induced differentiation of human lung fibroblasts to myofibroblasts is a key event in the pathogenesis of pulmonary fibrosis. Although the typical TGFβ signaling pathway involves the Smad family of transcription factors, we have previously reported that peroxisome proliferator-activated receptor-γ (PPAR-γ) ligands inhibit TGFβ-mediated differentiation of human lung fibroblasts to myofibroblasts via a Smad-independent pathway. TGFβ also activates the phosphatidylinositol 3 kinase/protein kinase B (PI3K/Akt) pathway leading to phosphorylation of Akt^S473^. Here, we report that PPAR-γ ligands, 2-cyano-3,12-dioxooleana-1,9-dien-28-oic acid (CDDO) and 15-deoxy-(12,14)-15d-prostaglandin J_2_ (15d-PGJ_2_), inhibit human myofibroblast differentiation of normal and idiopathic pulmonary fibrotic (IPF) fibroblasts, by blocking Akt phosphorylation at Ser473 by a PPAR-γ-independent mechanism. The PI3K inhibitor LY294002 and a dominant-negative inactive kinase-domain mutant of Akt both inhibited TGFβ-stimulated myofibroblast differentiation, as determined by Western blotting for α-smooth muscle actin and calponin. Prostaglandin A_1_ (PGA_1_), a structural analogue of 15d-PGJ_2_ with an electrophilic center, also reduced TGFβ-driven phosphorylation of Akt, while CAY10410, another analogue that lacks an electrophilic center, did not; implying that the activity of 15d-PGJ_2_ and CDDO is dependent on their electrophilic properties. PPAR-γ ligands inhibited TGFβ-induced Akt phosphorylation via both post-translational and post-transcriptional mechanisms. This inhibition is independent of MAPK-p38 and PTEN but is dependent on TGFβ-induced phosphorylation of FAK, a kinase that acts upstream of Akt. Thus, PPAR-γ ligands inhibit TGFβ signaling by affecting two pro-survival pathways that culminate in myofibroblast differentiation. Further studies of PPAR-γ ligands and small electrophilic molecules may lead to a new generation of anti-fibrotic therapeutics.

## Introduction

Idiopathic Pulmonary Fibrosis (IPF) is a progressive disease of unknown etiology that can result in respiratory failure [1], [2]. IPF is anatomically characterized by scarring of lung tissues owing to excessive deposition of extracellular matrix proteins (ECM). This excessive and uncontrolled deposition of ECM compromises normal lung function and structure [1], [3]. Fibroblasts are structural cells that show plasticity and ability to differentiate into myofibroblasts upon tissue injury or inflammation [4], [5]. Myofibroblasts are characterized by expression of alpha smooth muscle actin (αSMA), calponin and extracellular matrix (ECM) proteins including Type I and III collagen (Col1A1 and Col3A1), fibronectin and proteoglycan [4]. Deposition of ECM and other proteins produced by myofibroblasts plays an important role in normal physiologic processes such as wound healing. However, in pathologic conditions such as IPF, myofibroblasts accumulation and matrix deposition is excessive leading to scarring [1], [2].

Transforming Growth Factor β (TGFβ) is a pleiotropic cytokine that promotes myofibroblast differentiation and plays a major role in both wound healing and fibrosis [4], [6], [7]. Binding of active TGFβ to its receptor triggers several signaling pathways [8], [9], including the well-characterized Smad pathway and the PI3K/Akt (phosphotidylinositol-3-kinase/Protein Kinase B/PKB) pathway [7], [10], [11]. PI3K activates Akt by phosphorylation at two sites, Thr^308^ and Ser^473^ [10]. Once active, Akt functions as a serine/threonine kinase, which is involved in multiple cellular processes including cell proliferation, inflammation, survival and glucose metabolism. Akt is inactivated by a specific phosphatase enzyme, PTEN (the phosphatase and tensin homologue deleted on chromosome 10) [12]. Multiple upstream signaling events can activate Akt via PI3K. In fetal lung fibroblasts [13], and other organs [14], [15], TGFβ activates Akt signaling via p38 Mitogen Activated Protein Kinase (MAPK) and Focal Adhesion Kinase (FAK) [16], [17]. FAK is a non-receptor protein tyrosine kinase that is phosphorylated in response to integrin clustering and growth factor-mediated migration [18]. FAK is rapidly recruited to the focal adhesion upon integrin clustering [19], and is subsequently activated by phosphorylation at Tyr397. Increase in phosphorylation of FAK^Y397^ correlates with its increased catalytic activity [20], [21] and is required for the recruitment of p85, a regulatory subunit of PI3K [22]. In fetal lung fibroblasts, FAK is involved in myofibroblast differentiation via TGFβ, adhesion and β1-integrin mediated pathways [17], [23]. Moreover, it has been implicated as an upstream activator of Akt and may thus contribute to fibrogenesis [24], [25], [26].

Designing any effective therapy for pulmonary fibrosis requires precise understanding of the signaling events that are responsible for myofibroblast differentiation. We have previously reported that ligands of peroxisome proliferator-activated receptor-γ (PPAR-γ) suppress TGFβ-induced myofibroblast differentiation [27], [28] in a Smad-independent manner. PPAR-γ is a ligand-activated nuclear receptor which has been extensively studied for its involvement in adipogenesis, insulin sensitization, differentiation, proliferation [29], and more recently, for its anti-inflammatory and anti-fibrotic activities [29], [30], [31], [32], [33]. Typically upon ligand binding, PPAR-γ heterodimerizes with Retinoid Acid Receptor (RXR) and binds to PPAR-Response-Elements (PPRE) on target genes resulting in a transcriptional response [29]. Three of the main classes of PPAR-γ ligands include; Thiazolidinediones or TZDs (e.g. Rosiglitazone), Prostaglandins (e.g. 15d-PGJ_2_: 15-deoxy-Δ12, 14 -Prostaglandin J_2_) and, Triterpenoids (e.g. CDDO: 2-cyano-3,12-dioxoolean-1,9-dien-28-oic-acid). We and others have shown that PPAR-γ ligands inhibit TGFβ-mediated trans-differentiation of human lung fibroblasts to myofibroblasts, and this mechanism is largely PPAR-γ independent [32], [33]. The exact molecular mechanism of action of PPAR-γ ligands remains poorly understood.

Here, we report that the PI3K/Akt and FAK pathways are crucial for fibroblast to myofibroblast differentiation of normal and diseased primary human lung fibroblasts. Our data identify a novel mechanism by which PPAR-γ ligands inhibit TGFβ-induced fibroblast to myofibroblast differentiation and suggest that it may be possible to develop small molecule Akt and FAK inhibitors for use as novel anti-fibrotic therapeutics.

## Results

### TGFβ-Stimulated Myofibroblast Differentiation of Primary Human Lung Fibroblasts Requires the Phosphatidylinositol 3-Kinase Pathway

**Figure 1:**
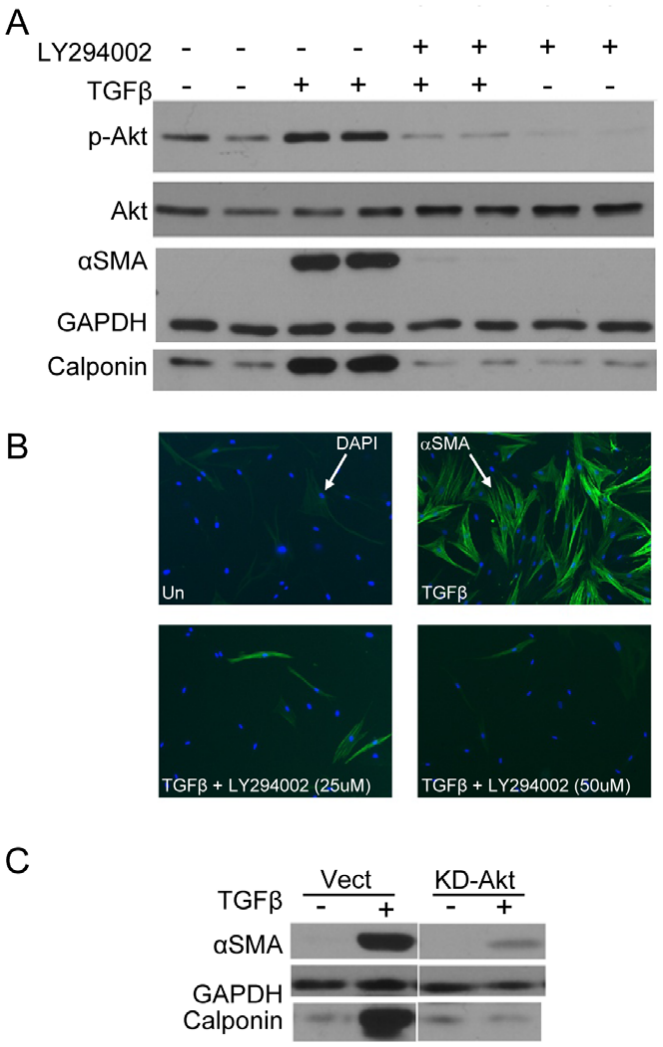
Inhibition of PI3K-Akt pathway by LY294002 inhibits myofibroblast differentiation. Primary HLFs were treated with the PI3K inhibitor LY294002 (50µM) followed by TGFβ (5ng/ml) for 48 hours and *A*, immunoblots were performed to detect expression of the indicated proteins, and *B*, immunofluorescence for αSMA (green) was performed to assess the effects of PI3K inhibition on TGFβ-induced myofibroblast differentiation. DAPI (blue) was used to visualize nuclei. *C*, HLF cells were transfected with an empty vector or a dominant negative kinase-dead (KD) Akt construct, treated with TGFβ, and assayed for myofibroblast differentiation by Western blot. Protein lysates from all the indicated samples were electrophoretically separated on the same gel, and representative lanes from a single experiment are shown here. These data indicate that a functional PI3K-Akt pathway is essential for the TGFβ-induced myofibroblast differentiation in primary human lung fibroblast.

TGFβ is known to induce cell-type specific actions that are context-specific and microenvironment-dependent [9]. We previously reported that inhibition of TGFβ-stimulated myofibroblast differentiation of primary human lung fibroblast (HLF) by PPAR-γ ligands was largely Smad-independent [33]. To investigate potential non-Smad signaling pathways, we examined whether TGFβ drives myofibroblast differentiation via the PI3K/Akt pathway. Primary HLF cells were treated with TGFβ in presence or absence of LY294002, a highly selective and potent inhibitor of PI3K. Following treatment, whole-cell lysates were subjected to specific Western blot analyses. As evident in Fig 1A, TGFβ potently induced phosphorylation of Akt. TGFβ was also able to induce myofibroblast differentiation as determined either by Western blot analysis (Fig 1A) or indirect immunofluorescence (Fig 1B) for αSMA and calponin. Pretreatment of cells with LY294002 markedly reduced TGFβ-induced phosphorylation of Akt (Fig 1A) and almost completely inhibited myofibroblast differentiation (Fig 1A and 1B). LY294002 treatment alone significantly reduced basal level of Akt phosphorylation and expression of calponin (Fig 1A). These results indicate that TGFβ induces phosphorylation of Akt and myofibroblast differentiation via the PI3K pathway in primary HLF.

To investigate if functional Akt kinase is required for TGFβ-induced myofibroblast differentiation, primary HLF cells transfected with either an empty vector or a dominant negative kinase-dead Akt (KD-Akt^K179A^) plasmid that encodes a mutant form of Akt [34] and lacks kinase activity [35]. Overexpression of the KD-Akt^K179A^ markedly inhibited TGFβ-induced expression of αSMA and calponin (Fig 1C) indicating that the intact kinase domain of Akt is essential for TGFβ-induced myofibroblast differentiation. These results firmly establish that TGFβ induces myofibroblast differentiation of primary human lung fibroblasts via a PI3K-Akt-dependent mechanism.

### PPAR-γ Ligands Block TGFβ-induced Phosphorylation of Akt in a Dose-Dependent Manner

After establishing that Akt activity is essential for TGFβ-induced myofibroblast differentiation, we examined whether PPAR-γ ligands CDDO and 15d-PGJ_2_ inhibit Akt phosphorylation. Primary HLF cells were treated with TGFβ alone or in combination with varying pharmacological concentrations of PPAR-γ ligands. The efficacy of PPAR-γ ligands to repress TGFβ-induced Akt phosphorylation at various concentrations was assessed by Western blot analysis.

**Figure 2:**
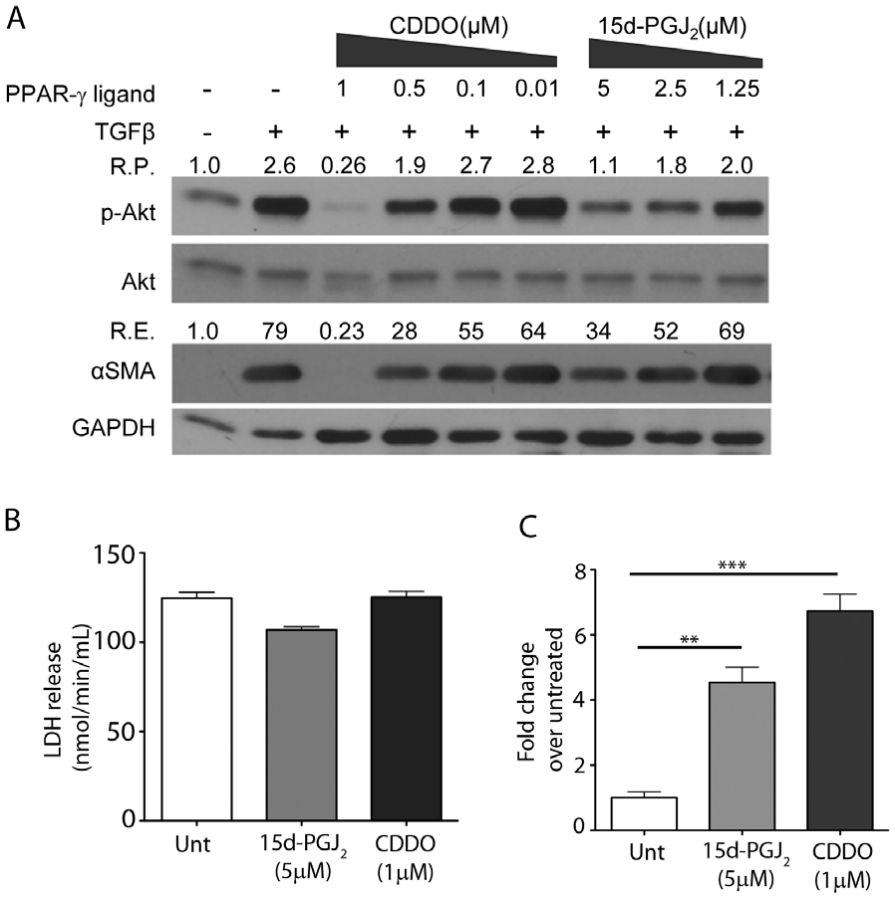
PPAR-γ ligands inhibit TGFβ-induced phosphorylation of Akt and myofibroblast differentiation in a dose-dependent manner. *A*, Primary HLFs were grown until 70–80% confluent, serum starved for 24 hours and treated with the indicated concentrations of PPAR-γ ligands for 48 hours. Total cell lysates were prepared, and subjected to SDS-PAGE followed by immunoblotting. The blot was probed with antibodies against phospho-Akt^S473^, stripped and probed to detect total Akt, αSMA and loading control GAPDH. The relative changes in the ratio of phospho-Akt^S473^/total Akt (R.P.) and relative changes in the expression of αSMA/GAPDH (R.E.) are as indicated in the figure. The experiment was performed in triplicate and a representative blot is shown here. *B*, LDH release does not increase in response to 15d-PGJ_2_ or CDDO. Primary human lung fibroblasts were treated with either 5 µM 15d-PGJ_2_ or 1µM CDDO for 72 hours and LDH release was measured (nmol/min/mL). *C*, Primary human lung fibroblasts were transfected with a PPRE luciferase reporter and a CMV β-galactosidase construct. Cells were treated with either 5µM 15d-PGJ_2_ or 1 µM CDDO for 48 hrs and luciferase activity was measured. Background was subtracted and data normalized to β-galactosidase transfection efficiency and reported as fold induction of luciferase units over the untreated samples. These data represent three independent experiments (mean ± S.E. shown, **p≤0.01, *** p≤0.001, compared to untreated).

Both CDDO and 15d-PGJ_2_ inhibited Akt phosphorylation as well as myofibroblast differentiation (Fig 2), but CDDO was about five times more potent than 15d-PGJ_2_ (IC_50_ of 0.5 µM and 2.5 µM, respectively). The potency of PPAR-γ ligands to inhibit TGFβ-induced myofibroblast differentiation correlates extremely well with their relative efficacy in inhibiting Akt phosphorylation under the same physiological conditions (Fig 2 and [33]).Taken together, our results obtained thus far demonstrate that a functional PI3K-Akt pathway is essential for TGFβ-driven myofibroblast differentiation, and PPAR-γ ligands target this pathway through inhibition of Akt^S473^ phosphorylation.

Since Akt is involved in the cell survival pathway next, we examined if 15d-PGJ_2_ and CDDO are cytotoxic at the concentrations used in these experiments. Neither 15d-PGJ_2_ ([27] and Fig 2B) nor CDDO (Fig 2B) were found to be cytotoxic as measured by LDH release assay. Additionally, we confirmed that 15d-PGJ_2_ and CDDO both were able to induce PPAR-γ-dependent transcription as measured by their ability to induce PPRE-luciferase (PPAR-γ Response Elements-luciferase) (Fig 2C and [27]).

### Inhibition of TGFβ-Stimulated Activation of Akt by PPAR-γ Ligands is Independent of PPAR-γ Activity

Typically, PPAR-γ agonists bind to the ligand binding site of PPAR-γ, causing its nuclear translocation and resulting in a transcriptional response at target genes [10]. However, we [27], [28], [33] and others [32], have shown that PPAR-γ ligands also have effects that are independent of PPAR-γ activity. To examine if inhibition of Akt phosphorylation by PPAR-γ ligands was dependent on the functional activity of PPAR-γ, we used a pharmacological approach involving a PPAR-γ antagonist GW9662, an irreversible PPAR-γ antagonist that covalently binds to the active site of PPAR-γ [36].

**Figure 3:**
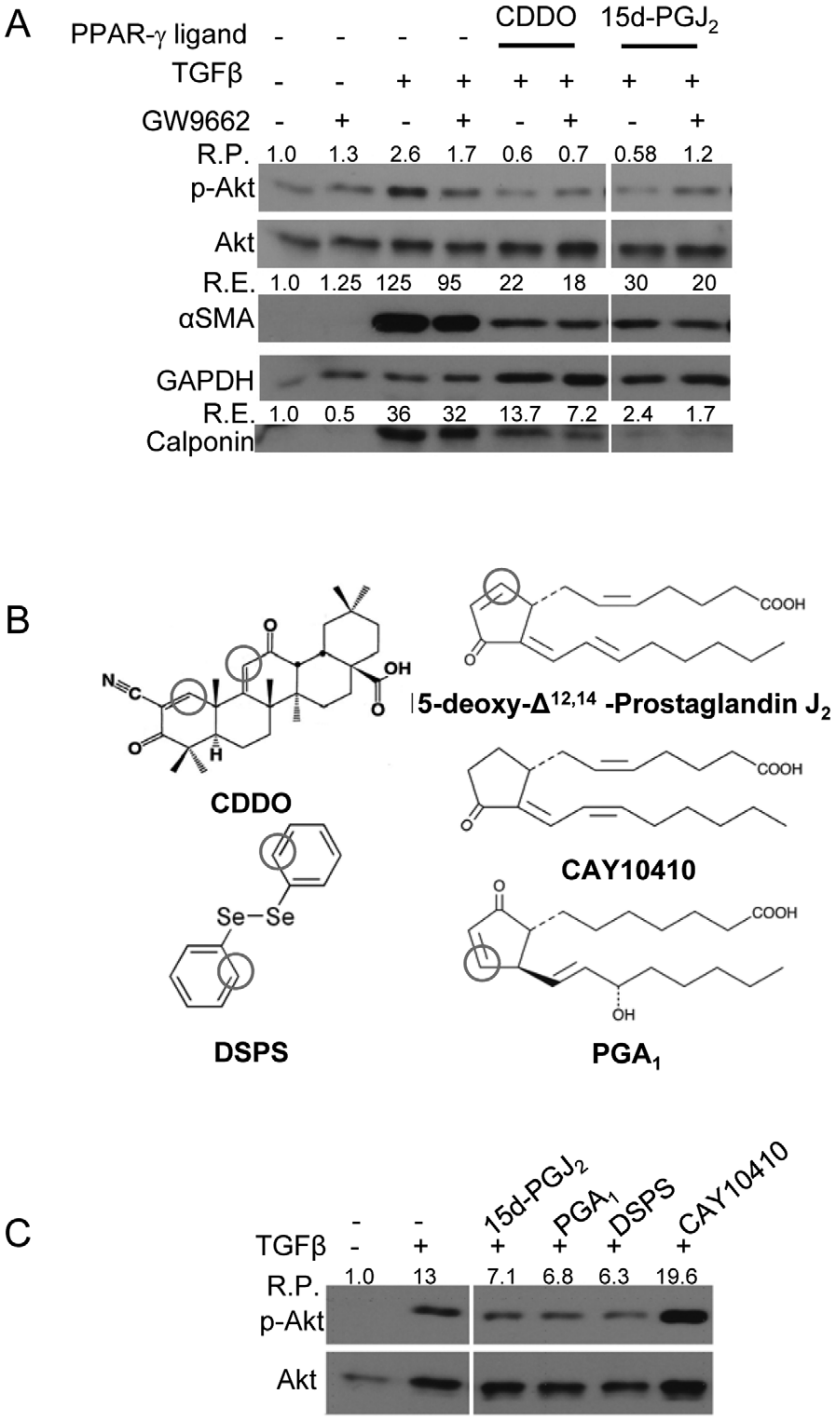
PPAR-γ ligands inhibit TGFβ-induced Akt phosphorylation and myofibroblast differentiation in a PPAR-γ-independent but electrophilic carbon-dependent manner. HLF cells were treated with indicated compounds and/or TGFβ (5ng/ml) for 48 hours. Immunoblots were performed to assess the expression of indicated proteins. Protein lysates from all the indicated samples were electrophoretically separated on the same gel, and representative lanes from a single experiment are shown here. *A*, the ability of PPAR-γ ligands (CDDO (1µM) and 15d-PGJ_2_ (5µM)) to reduce p-Akt was not altered upon GW9662-mediated inhibition of PPAR-γ. GW9662 (5µM) inhibits PPAR-γ activity by a covalent bond formation with PPAR-γ protein [36]. R.P. indicates relative changes in Akt phosphorylation compared to control sample, and R.E., relative changes in expression compared to control sample. *B*, PPAR-γ ligands contain electrophilic carbons. Here, positions of the electrophilic carbons in the structures of the compounds are marked. CAY10410 and PGA_1_ are structural analogues of 15-d-PGJ_2_. PGA_1_ has an electrophilic center but CAY10410 does not. DSPS, like CDDO, has two electrophilic centers. Cells were pre-treated with CAY10410 (5µM), PGA_1_ (10µM) and DSPS (10µM) for 30 minutes *C*, only compounds with an electrophilic carbon are able to reduce Akt phosphorylation, indicating that presence of an electrophilic carbon is essential for the observed reduction in the phosphorylation of Akt. All the experiments were performed in triplicate and representative images are shown here.

Primary HLF were pretreated with GW9662 followed by treatment with PPAR-γ ligands and TGFβ. After 48 hours, total protein lysate was subjected to Western blot analysis to assess the level of changes in Akt phosphorylation. TGFβ alone increased phosphorylation of Akt while addition of CDDO or 15d-PGJ_2_ blocked TGFβ-induced Akt phosphorylation (Fig 3A). However, treatment with GW9662 did not restore either TGFβ-induced phospho-Akt levels in PPAR-γ-ligand-treated cultures or myofibroblast differentiation (Fig 3A) suggesting that CDDO and 15d-PGJ_2_ block TGFβ-induced Akt phosphorylation in a PPAR-γ-independent mechanism.

### The Electrophilic Center Present in PPAR-γ Ligands is Critical to Their Ability to Block TGFβ-induced Phosphorylation of Akt

CDDO and 15d-PGJ_2_ contain electrophilic carbons (Fig 3B) that can modify sulfhydryl groups in proteins via the ‘Michael addition reaction’ [37], [38]. To determine whether the ability of PPAR-γ ligands to inhibit TGFβ-induced Akt phosphorylation is dependent on the presence of an electrophilic carbon, we used two different structural analogues of 15d-PGJ_2_, prostaglandin A_1_ (PGA_1_), which has an electrophilic center, and CAY10410, which does not have an electrophilic carbon but binds to PPAR-γ and activates ligand-dependent transcription. Additionally, we used another potent electrophilic compound, diphenyl diselenide (DSPS), which, like CDDO, is structurally different from 15d-PGJ_2_ and has two electrophilic carbons (Fig 3B). Interestingly, only the compounds with electrophilic centers, PGA_1_ and DSPS, reduced TGFβ-driven phosphorylation of Akt, while CAY10410, did not (Fig 3C). Based on these results, we concluded that the electrophilic carbons present in the structures of CDDO and 15d-PGJ_2_ have an essential role in blocking TGFβ-mediated activation of Akt.

### PPAR-γ Ligands Inhibit TGFβ-induced Phosphorylation of Akt in a Transcription-Independent Manner

**Figure 4:**
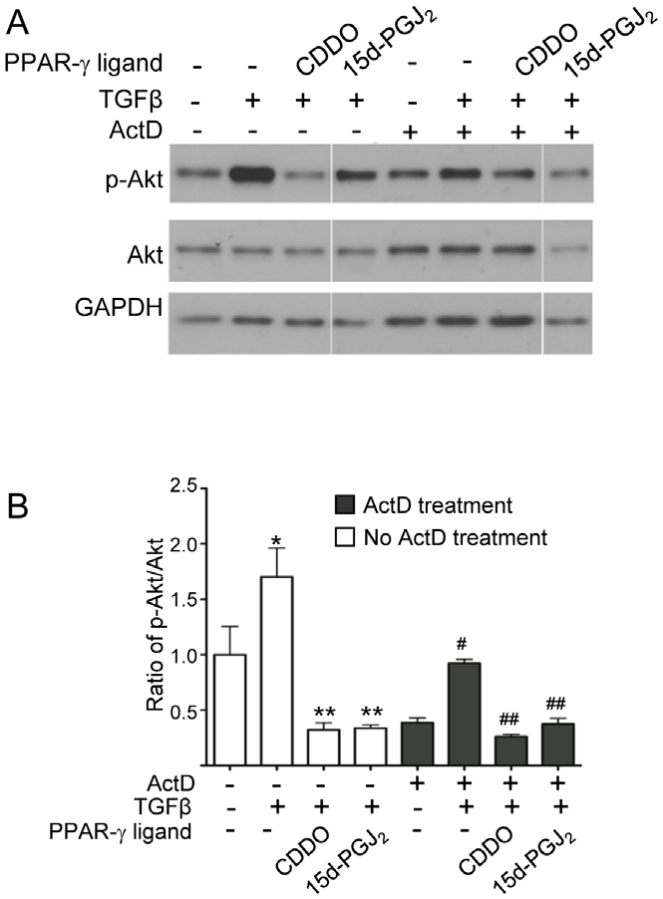
Actinomycin D partially inhibits TGFβ-induced phosphorylation of Akt but not PPAR-γ ligand-mediated inhibition of Akt phosphorylation. Primary HLFs were pre-treated with transcription inhibitor ActD (1µg/ml), followed by PPAR-γ ligands (CDDO (1µM) and 15d-PGJ_2_ (5µM)) and TGFβ (5ng/ml). *A*, Immunoblots were performed to detect levels of indicated proteins upon inhibition of transcription. The experiment was performed with triplicate samples. Protein lysates from all the indicated samples were electrophoretically separated on the same gel, and representative lanes from a single experiment are shown here. *B*, The triplicate samples were measured by densitometry and the ratio of phospho to total Akt was determined and normalized to untreated and bar graphs were plotted. The significance was calculated by one way ANOVA. For samples without ActD treatment (open bars), * indicates difference (P≤0.05) over untreated and ** indicates difference (P≤0.05) over TGFβ-treated samples. For ActD-treated samples (black bars), # indicates difference (P≤0.05) over untreated and ## indicates difference (P≤0.05) over TGFβ-treated samples.

Because the effects of CDDO and 15d-PGJ_2_ are PPAR-γ independent, we hypothesized that their effect would be independent of transcription as well. To investigate if PPAR-γ ligands require *de novo* transcription for their inhibition of Akt phosphorylation we treated primary HLF cells with a transcription inhibitor Actinomycin D (ActD), followed by the PPAR-γ ligands and TGFβ. Western blot analysis was performed to detect phospho-Akt^S473^ and total Akt and the ratio of phospho-Akt^S473^/Akt was calculated. ActD partly inhibited TGFβ-induced phosphorylation of Akt (Fig 4). This suggests that TGFβ activates Akt kinase in a transcriptionally-dependent mechanism. However, inhibition by CDDO and 15d-PGJ_2_ remained unaltered even in the presence of ActD (Fig 4A, and Fig 4B, black bars) suggesting that these compounds directly inhibit Akt kinase activity or upregulate a phosphatase in a post-translational mechanism that is independent of *de novo* transcription.

### PPAR-γ Ligands Block TGFβ-Stimulated Phosphorylation of Akt by Inhibiting FAK but not PTEN and MAPK-p38 Phosphorylation

To investigate proteins that could potentially phosphorylate or dephosphorylate Akt, we performed a time-course of action of PPAR-γ ligands and TGFβ on the phosphorylation of PTEN, p38-MAPK and FAK.

**Figure 5:**
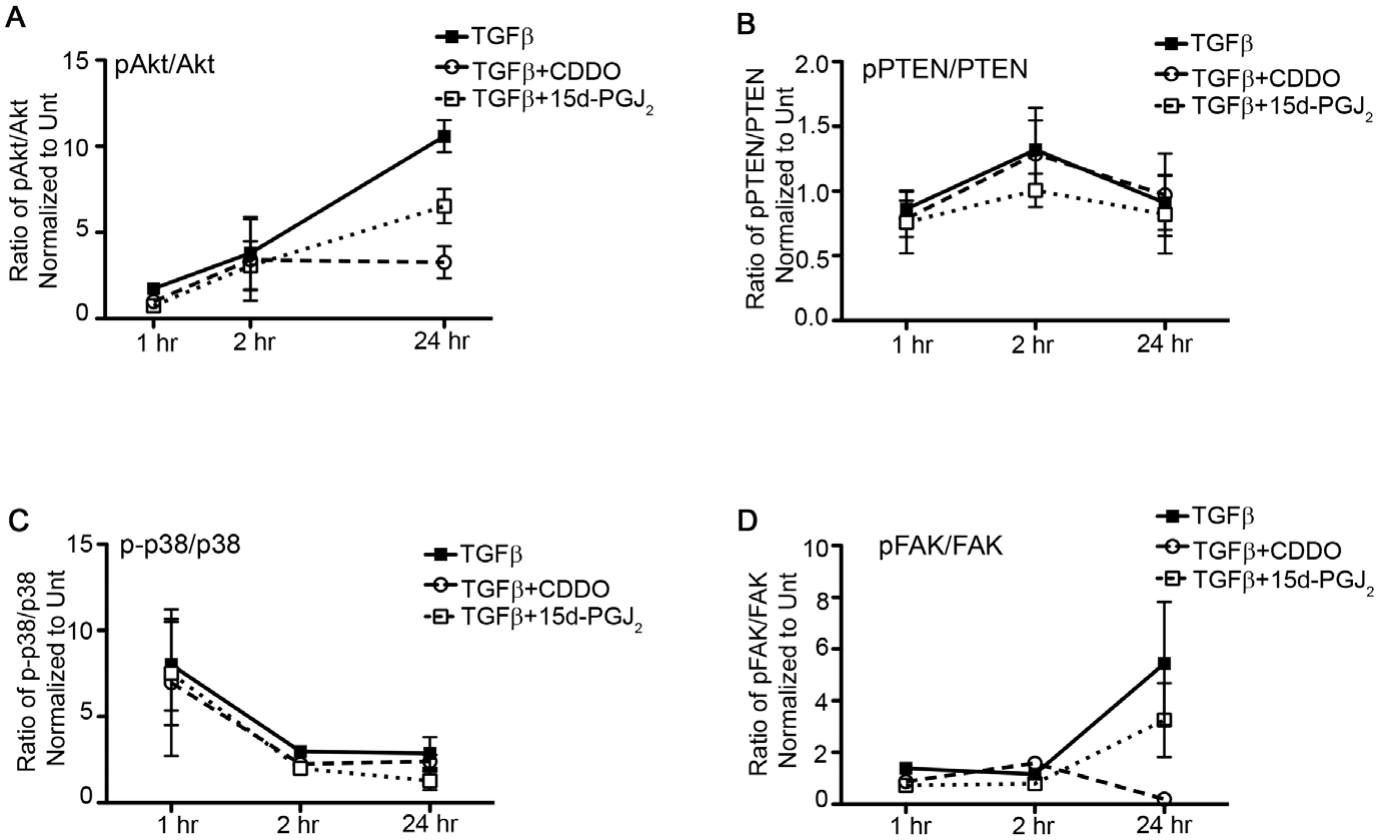
PPAR-γ ligands inhibit TGFβ-induced phosphorylation of Akt and FAK but not MAPK-p38 and PTEN. Primary HLFs were pretreated with PPAR-γ ligands; CDDO (1µM) and 15d-PGJ_2_ (5µM) followed by TGFβ (5ng/ml). Cells were harvested and lysates analyzed by immunoblots at the indicated time. The ratio of phospho-protein to total protein was measured by densitometric analysis and normalized to untreated cells (untreated = 1.0). TGFβ-induced phosphorylation of *A*, Akt^S473^ and *D*, FAK^Y397^ was inhibited significantly by the PPAR-γ ligands but the phosphorylation of *B*, PTEN^T308^ and *C*, p38-MAPK ^T180/Y182^ was not affected. The statistical significance over TGFβ-treatment alone was calculated either by one way ANOVA on triplicate samples (A, B and C) or using unpaired t-test on duplicate samples (D) and is indicated as * where P≤0.05.

First, we investigated the time-course of TGFβ-mediated phosphorylation of Akt and its repression by CDDO and 15d-PGJ_2_ (Fig 5A). Next, we examined changes in phosphorylation of PTEN, which is a negative regulator of Akt phosphorylation. PTEN is more stable but less active when it is phosphorylated at T308, and less stable but more active when is dephosphorylated at the same site [39]. If PPAR-γ ligands inhibit Akt phosphorylation via upregulation of phosphatase activity of PTEN, we would expect to see a decrease in the ratio of phospho-PTEN^T308^ to total PTEN. We found that although TGFβ slightly increased levels of phospho-PTEN after two hours (Fig 5B, filled squares), treatment with PPAR-γ ligands was unable to cause any significant deviation in the ratio of phospho-PTEN to total PTEN as compared to the TGFβ alone-treated samples (Fig 5B, open squares and circles).

It has been reported that TGFβ induces p38-MAPK in some cell types [17]. We also observed an increase in the phospho-p38^T180/Y182^ to total p38 ratio in response to TGFβ but, phosphorylation of TGFβ-induced p38 was not reduced upon treatment with either CDDO or 15d-PGJ_2_ (Fig 5C), indicating that the mode of action of PPAR-γ ligands is likely independent of p38-MAPK activity. Finally, we examined the effect of PPAR-γ ligands on FAK^Y397^ phosphorylation, which is required for FAK kinase activity. We observed TGFβ-induced phosphorylation of FAK repressed by both the PPAR-γ ligands 24 hours after the treatment (Fig 5D). CDDO inhibited FAK^Y397^ phosphorylation more potently than 15d-PGJ_2_ (Fig 5D). Since PPAR-γ ligands inhibit FAK phosphorylation but do not change either p38-MAPK or PTEN phosphorylation, we suggest that PPAR-γ ligands inhibit Akt pathway by inhibiting the upstream FAK kinase.

### Pharmacological Inhibition of FAK Activity Inhibits the PI3K-Akt Pathway and Myofibroblast Differentiation

**Figure 6:**
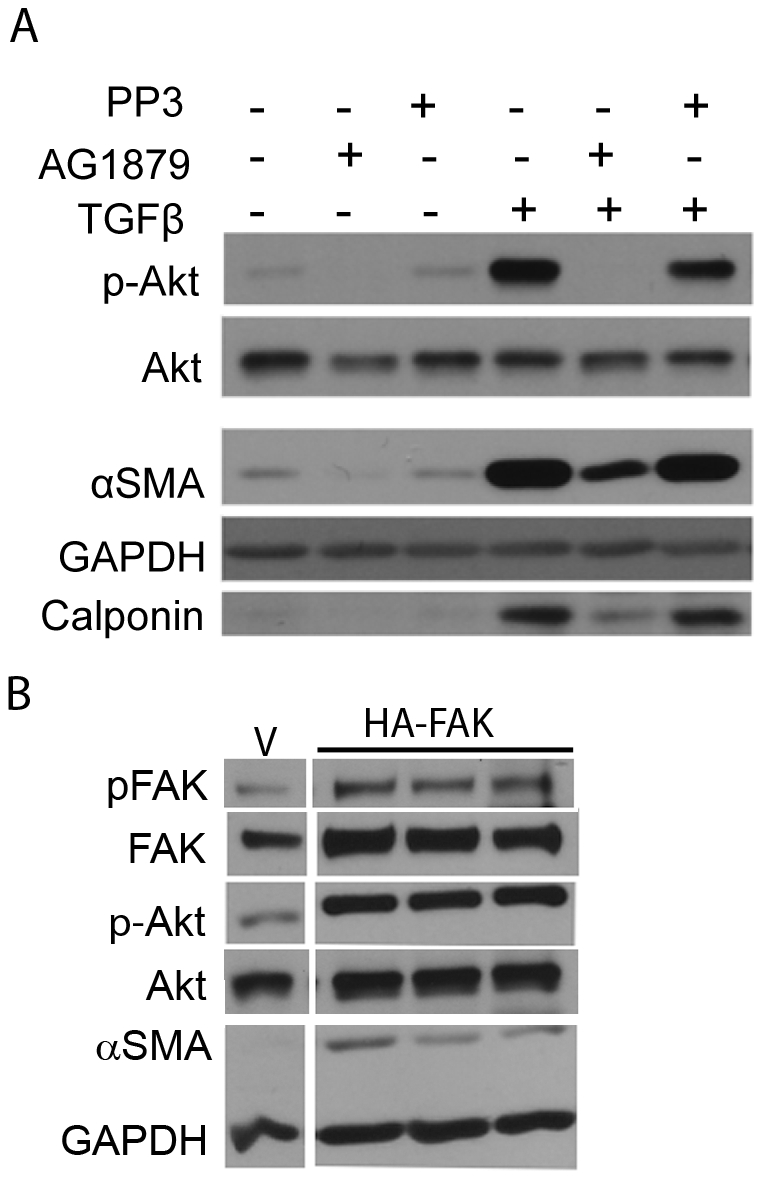
Pharmacological inhibition of FAK activity inhibits the PI3K-Akt pathway and myofibroblast differentiation. *A*, Primary HLFs were treated in presence or absence of TGFβ and 10µM specific Src-FAK kinase inhibitor AG1879 (PP2) or its analogue, PP3, that does not inhibit FAK activity and immunoblots were performed to analyze expression of the indicated proteins. The FAK inhibitor AG1879, but not its analogue PP3, inhibited TGFβ-induced phosphorylation of FAK^Y397^ and Akt^S473^ and reduced myofibroblast differentiation as determined by expression of αSMA and calponin. *B*, HLF cells were transfected with the empty vector (V) or FAK overexpressing construct (HA-FAK) and assayed for myofibroblast differentiation by Western blot. Protein lysates from all the indicated samples were electrophoretically separated on the same gel, irrelevant lanes excluded and representative lanes from a single experiment are shown here. These data indicate that FAK overexpression induces myofibroblast differentiation of primary human lung fibroblasts.

To determine whether TGFβ-mediated myofibroblast differentiation mediated through PI3K-Akt signaling also requires FAK activity in our cell strain, we treated primary HLF cells with a FAK-kinase inhibitor (AG1879) and its inactive analogue, PP3. Western blot analysis was used to measure phospho-FAK^Y397^, total FAK, phospho-Akt^S473^, total Akt, αSMA, calponin and GAPDH. We observed that inhibition of FAK inhibited not only Akt phosphorylation but also myofibroblast differentiation (Fig 6). Although, inhibition of FAK inhibited αSMA expression, we did not observe complete inhibition of expression of αSMA under the conditions tested. These results indicate that FAK activity is important for TGFβ-mediated Akt activation and myofibroblast differentiation of primary HLF.

Next, we used a complimentary genetic approach to ascertain involvement of FAK in myofibroblast differentiation of primary HLFs. We found that over-expression of FAK resulted in marked up-regulation of Akt phosphorylation and myofibroblast differentiation (Fig 6B).

### PPAR-γ Ligands Inhibit Myofibroblast Differentiation of Primary IPF Fibroblasts by Inhibiting FAK and PI3K-Akt Pathways

**Figure 7:**
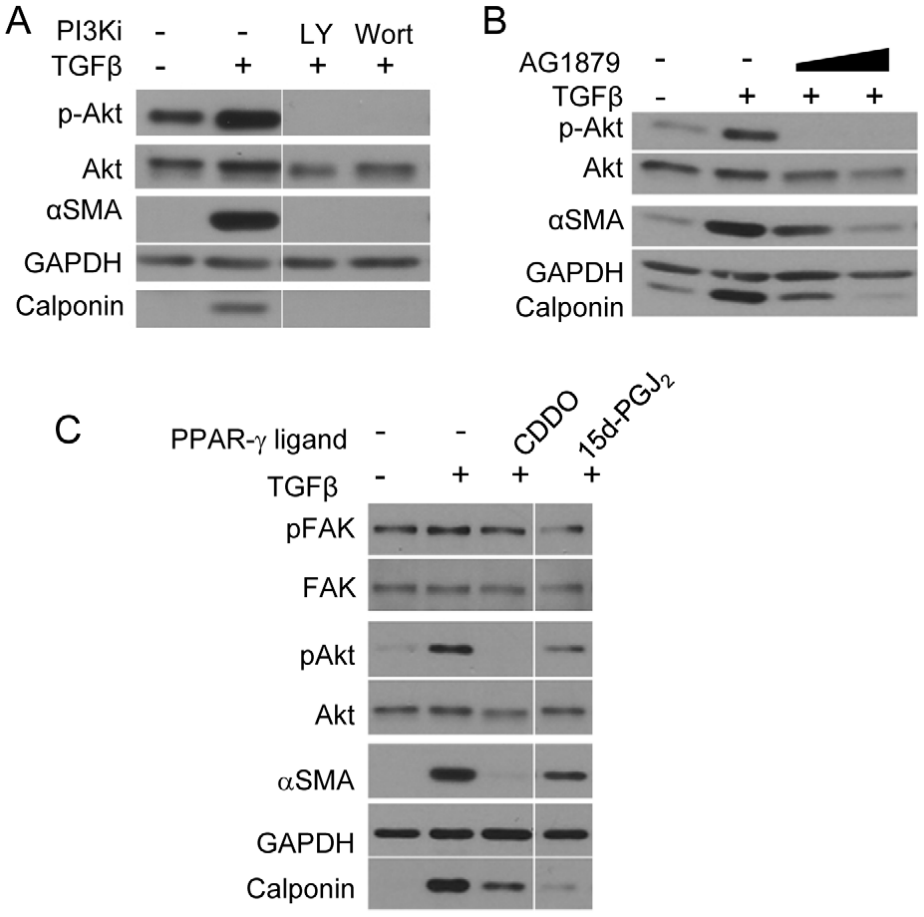
PPAR-γ ligands block myofibroblast differentiation of primary human IPF fibroblasts. Primary IPF fibroblasts were treated with either *A*, two PI3K inhibitors LY294002 (50µM) or wortmannin (100nM) or *B*, a Src-FAK inhibitor AG1879 (10 and 20µM) or *C*, CDDO (1µM) or 15d-PGJ_2_ (5µM) followed by TGFβ (5ng/ml) for 48 hours. Protein lysates were prepared and immunoblots were performed to detect expression levels of the indicated proteins. The experiment was performed in triplicate on three independent IPF fibroblast strains. Protein lysates from all the indicated samples were electrophoretically separated on the same gel, and representative lanes from one representative set of data are shown here. These data confirm that CDDO and 15d-PGJ_2_ inhibit both, FAK and PI3K-Akt pathways to inhibit TGFβ-induced myofibroblast differentiation of primary IPF fibroblasts.

To ascertain the therapeutic potential of PPAR-γ ligands we examined their ability to suppress Akt and FAK in *bona fide* diseased primary lung fibroblasts obtained from patients with IPF. First, we investigated whether TGFβ induced myofibroblast differentiation of IPF fibroblasts via activation of Akt and FAK pathways. IPF fibroblasts were treated with two highly potent and selective inhibitors of PI3K pathway, LY294002 and wortmannin (Fig 7A) or increasing concentrations of AG1879 (PP2), a FAK inhibitor (Fig 7B) followed by TGFβ. Inhibition of both the pathways potently blocked Akt phosphorylation and myofibroblast differentiation (Fig 7A and B). Finally, we treated IPF fibroblasts with CDDO and 15d-PGJ_2_ followed by TGFβ. These data firmly establish that CDDO and 15d-PGJ_2_ are both capable of blocking myofibroblast differentiation via Akt and FAK pathways (Fig 7C) as measured by the expression of αSMA and calponin.

## Discussion

Idiopathic pulmonary fibrosis (IPF) can lead to respiratory failure and death due to deteriorating respiratory function [1], [4]. There are currently few if any effective therapies. Therefore, novel antifibrotic drugs are urgently needed for the treatment of IPF and other scarring diseases.

PPAR-γ ligands are emerging as exciting potential therapeutics for inflammatory and fibrotic and other diseases [29], [40], [41]. PPAR-γ activation induces adipogenesis and differentiation, and represses inflammation [27], [29], [33]. PPAR-γ ligands including rosiglitazone and other members of TZD family are used for the treatment of type II diabetes [29]. Clinical trials for CDDO are in progress, and it has been found to be orally active for the treatment of solid tumors and lymphoma [42]. However, the mechanisms of anti-fibrotic actions of PPAR-γ ligands remain poorly understood. Therefore, we investigated the underlying molecular pathways targeted by distinct PPAR-γ ligands to explore the potential of PPAR-γ agonists as anti-fibrotic therapies.

Our previous work identified CDDO, a novel PPAR-γ ligand, as a potent anti-fibrotic agent of TGFβ-driven pro-fibrotic activity *in vitro* [27], [33]. TGFβ induces lung fibrosis *in vivo* [43] and also stimulates phosphorylation of Akt in animal models [44] and other human organs [10], [45]. Studies in fetal lung fibroblasts demonstrate the role of TGFβ-induced Akt pathway in myofibroblast differentiation [13]. Here, we report that in both normal and IPF primary human lung fibroblasts, PPAR-γ ligands potently block myofibroblast differentiation via a PPAR-γ-independent mechanism by targeting the TGFβ-induced PI3K-Akt pathway involving FAK.

We investigated the role of PI3K-Akt pathway in TGFβ-stimulated myofibroblast differentiation using LY294004, a specific PI3K activity inhibitor, and by using a kinase-dead (KD-Akt) construct of Akt. Both LY294002 and the Akt mutant strongly blocked TGFβ-stimulated myofibroblast differentiation, confirming the central role of PI3K-Akt pathway in TGFβ-mediated myofibroblast differentiation in adult human normal and “diseased” IPF lung fibroblasts (Fig 1 and 7). Although, CDDO [46], [47] and 15d-PGJ_2_ [48] have been reported to reduce Akt phosphorylation in some studies, their mechanism of reduction of TGFβ-induced myofibroblast differentiation through Akt pathway is not yet reported. Here, we show that the suppression of TGFβ-induced phosphorylation of Akt^S473^ is the central mechanism of action of CDDO and 15d-PGJ_2_ that leads to their anti-fibrotic activity. CDDO suppresses TGFβ-induced phospho-Akt more potently than 15d-PGJ_2_, which correlates very well with the abilities of CDDO and 15d-PGJ_2_ to reduce TGFβ-induced myofibroblast differentiation (Fig 2). Compared to CDDO and 15d-PGJ_2_, rosiglitazone was relatively poorly effective at inhibiting TGFβ-induced Akt phosphorylation (data not shown). Interestingly, Kilter et al. reported that rosiglitazone facilitates rephosphorylation of Akt in rat myocardiocytes [49]. We and others have reported that rosiglitazone has some anti-fibrotic effects *in vitro* [27], [32], [33], [50], but is much less potent than either CDDO or 15d-PGJ_2_. If rosiglitazone indeed facilitates re-phosphorylation of Akt, then it would result in a pro-fibrotic response that undercuts its anti-fibrotic effects, suggesting that rosiglitazone is not an optimal choice for treating fibrotic lung diseases. We did not investigate rosiglitazone further in this study.

CDDO and 15d-PGJ_2_ have electrophilic properties that rosiglitazone does not, and we have previously identified the electrophilic centers as important in the antifibrotic activity of these compounds. Building on these observations, here we demonstrate that the PPAR-γ-independent effects of CDDO and 15d-PGJ_2_ on Akt phosphorylation are dependent on the electrophilic properties (Fig 3). These observations offer an additional possibility that CDDO and 15d-PGJ_2_ could directly bind to the active site of a signaling molecule involved in the Akt pathway. One study has shown that biotinylated CDDO is capable of binding and thus inactivating the active site of PTEN [37], and 15d-PGJ_2_ is capable of inhibiting the activities of proteins through direct covalent modification [51], [52]. Further studies involving biochemical approaches should help us understand the exact nature of action of compounds involving electrophilic carbon. To generate newer anti-fibrotic therapeutics, further investigation of the electrophilic properties of PPAR-γ ligands and similar compounds is necessary.

**Figure 8:**
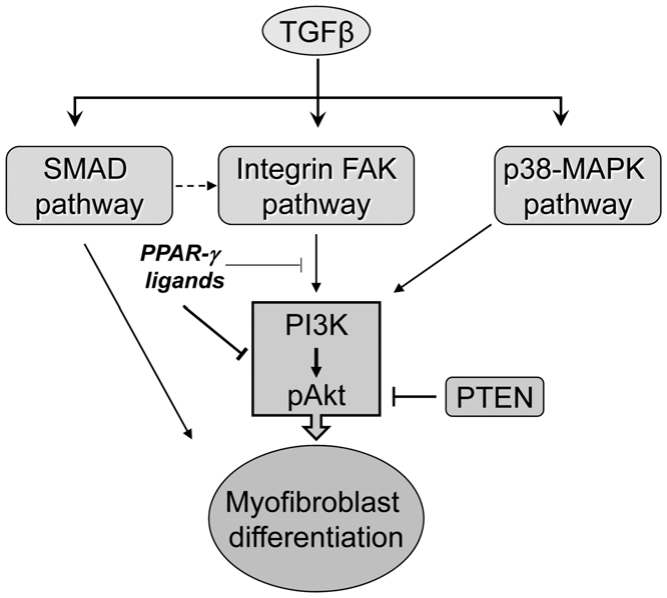
A proposed model showing the mechanism of action of electrophilic PPAR-γ ligands on TGFβ-induced myofibroblast differentiation. TGFβ induces myofibroblast differentiation by activating SMAD, FAK and PI3K-Akt pathways. However, PPAR-γ ligands inhibit the TGFβ-induced PI3K-Akt pathway, partly by targeting FAK induced activation of Akt.

Because CDDO and 15d-PGJ_2_ block Akt phosphorylation in the absence of new transcription (Fig 4), this suggests that CDDO and 15d-PGJ_2_ act to directly inhibit a kinase or activate a phosphatase that acts on Akt. We examined the role of three important upstream regulators of Akt phosphorylation: p38-MAPK, PTEN and FAK (Fig 8). We chose to examine p38-MAPK because it is previously known to be a part of the PI3K/Akt pathway [13] while other MAP kinases are involved in separate [53] or even antagonistic pathways [54]. TGFβ activates a number of signaling pathways including Smads, Akt and the MAPK-ERK, and while our data does not completely rule out that PPAR-γ ligands may act via other pathways, the ability of LY294002 and KD-Akt to almost completely block myofibroblast differentiation shows that the PI3K/Akt pathway is the most important pathway. Since TGFβ stimulates phosphorylation of p38-MAPK to activate myofibroblast differentiation by up-regulating Akt phosphorylation we examined involvement of p38-MAPK in primary HLF.

In agreement with a previous report [13], we determined that p38-MAPK is phosphorylated following TGFβ treatment of HLF (Fig. 5C), and MAPK inhibitor SB203580 reduced phosphorylation of Akt (data not shown). However, neither CDDO nor 15d-PGJ_2_ altered TGFβ-induced p38-MAPK phosphorylation, indicating that MAPK is likely not involved in inhibition of Akt by CDDO and 15d-PGJ_2_. We were also able to exclude PTEN as a major mediator of the effects of PPAR-γ ligands in HLFs. PTEN can inhibit the Akt pathway by dephosphorylating Akt, and PTEN phosphatase is itself activated by dephosphorylation at Thr^308^. Thus, if CDDO or 15d-PGJ_2_ blocked Akt phosphorylation via PTEN, we would expect increased PTEN phosphatase activity associated with increase in its dephosphorylation at T308. In fact, CDDO or 15d-PGJ_2_ do not change PTEN phosphorylation (Fig 5B). Interestingly, in human retinal epithelial cells, biotinylated CDDO (CDDO-Bt) binds to Cys124 within the active site of PTEN and inhibits the lipid phosphatase activity of PTEN *in vitro* [37]. If PTEN activity inhibition was an important mechanism in our system, we would expect levels of phospho-Akt to increase in presence of CDDO; instead we observed the opposite.

Multiple reports show that TGFβ stimulates autophosphorylation-dependent activation of focal adhesion kinase (FAK) [23], [55]. For example in CCL20 lung fibroblasts, Xia et al demonstrated that β1-integrin signaling upregulates FAK phosphorylation and its physical interaction with PI3K-p85 resulting in phosphorylation of Akt [24]. Although FAK is a widely accepted upstream regulator of Akt phosphorylation, it has been reported that FAK does not act upstream of Akt during TGFβ signaling in IMR90 human fetal lung fibroblasts [17]. However, it is widely accepted that integrins are able to activate TGFβ [56] and FAK [57], [58], both of which are involved in myofibroblast differentiation. Our results demonstrate that PPAR-γ ligands are able to inhibit phosphorylation of FAK and limit the TGFβ-mediated fibrotic response in adult primary HLFs (Fig 5D). We confirmed both in our normal (Fig 6) and IPF (Fig 7) primary human cell strains that blocking FAK activity inhibited not only phosphorylation of Akt, but also expression of αSMA, confirming that FAK indeed regulates myofibroblast differentiation under normal and diseased conditions through activation of Akt pathway. To establish the therapeutic potential of PPAR-γ ligands we treated fibroblasts obtained from IPF patients, in parallel, with PPAR-γ ligands, two PI3K inhibitors and a FAK inhibitor and confirmed that CDDO and 15d-PGJ_2_ potently block myofibroblast differentiation of not only normal HLF but also diseased IPF fibroblasts (Fig 7) via PI3K-Akt and FAK pathways. Although by using a complimentary genetic approach we confirmed that overexpression of FAK is capable of upregulating Akt phosphorylation and myofibroblast differentiation (Fig 6B), at this point, we cannot rule out an additional mechanism of action of PPAR-γ ligands that would result in reduction of Akt phosphorylation (Fig 8).

Current models of pulmonary fibrosis suggest that TGFβ, mechanical stress, or adhesion and integrin mediated activation of myofibroblast differentiation all contribute to upregulation of a fibrotic response. One very critical, central and shared event in all of these pathways involves upregulation of FAK activity, defined by its phosphorylation at Tyr397. It is conceivable that once TGFβ activates myofibroblast differentiation, the increased deposition of extracellular matrix proteins would cause additional mechanical stress on the cell surface leading to sustained and continual activation of FAK. Since FAK itself upregulates myofibroblast differentiation, once TGFβ initiates this process, sustained activation of FAK would be able to perpetuate the fibrotic response even in the absence of active TGFβ. Our work is the first report in any biological system demonstrating that PPAR-γ ligands reduce FAK activity by reducing FAK phosphorylation at Tyr397. Since FAK plays a cardinal role in myofibroblast differentiation, drugs that target the catalytic activity of FAK could be very valuable in the treatment of pulmonary fibrosis.

This study highlights a very important mechanism of action of CDDO and 15d-PGJ_2_ that involves down-regulation of PI3K-Akt pathway in both normal and IPF fibroblasts. Knowing that Akt is a central regulator of multiple cellular pathways including cell proliferation, cell cycle progression, inflammation and apoptosis [7], [10], interfering with the Akt pathway can have multiple cellular and organ-wide effects. Although, we have noted sustained basal activity of Akt in untreated cells, the nature of Akt activation is largely inducible and dependent on upstream signaling molecules. Therefore, the use of Akt-inhibition as a potential therapy for pulmonary fibrosis is a very novel and exciting concept.

Overall, we propose that certain PPAR-γ ligands have tremendous translational potential as therapeutics for pulmonary fibrosis by not only inhibiting Akt but also FAK activation. Future *in vivo* studies involving PPAR-γ ligands will be pivotal in exploring the promising potential of PPAR-γ ligands as therapeutics for pulmonary fibrosis as well as other scarring diseases.

## Materials and Methods

### Cells and reagents

Normal primary human lung fibroblasts (HLFs) were derived as previously described [33], grown in MEM supplemented with 10%FBS, L-Glutamine, antibiotic and antimycotic (Gibco, Carlsbad, CA) and used between passages 7–10. They were grown until 70–80% confluent and serum-starved for 24 hrs before treatment, unless otherwise mentioned. Primary human idiopathic pulmonary fibrotic (IPF) fibroblasts were derived from lung tissues obtained from patients with IPF undergoing wedge biopsy. A written informed consent was obtained from all the subjects in accordance with the University of Rochester Medical Center Institutional Review Board. Explant technique was used to isolate primary fibroblasts as described previously [33] and fibroblasts were used between passages 5–9 and maintained as described above.

### Treatments

PPAR-γ agonists were used at the following concentrations; 1µM of CDDO (NIH-RAID Program and Reata Pharmaceuticals, Dallas, TX), 5µM 15d-PGJ_2_ and 9,10-dihydro-15-deoxy-Δ12,14-PGJ2 (CAY10410) (Cayman Pharmaceuticals, Ann Arbor, MI). Ligands were dissolved in DMSO to make 10mM stock solution and diluted in serum-free media before treatment. GW9662 (Sigma, St. Louis, MO), 15-deoxy-Δ12,14-Prostaglandin A_1_ (PGA1) (Cayman Pharmaceuticals, Ann Arbor, MI) and diphenyl diselenide (DSPS) (Sigma, St. Louis, MO) were prepared in the same manner as described above. GW9662 (5µM) was added two hours prior to any other treatment. Human recombinant TGFβ1 (R&D Systems, Minneapolis, MN) was used at a final concentration of 5ng/ml. 50µM LY294002 (Cell Signaling Technology, Danvers, MA), 10 or 20µM AG1879 (4-Amino-5-(4-chlorophenyl)-7-(t-butyl)pyrazolo[3,4-d]pyrimidine, (PP2)) and its analogue, 4-Amino-7-phenylpyrazol[3,4-d]pyrimidine (PP3) (EMD Chemicals Inc. Gibbstown, NJ), 100nM wortmannin and, 1µg/ml Actinomycin D (Sigma, St. Louis, MO) were used to pretreat cells 30 min prior to TGFβ treatment.

### Western blots

Primary human lung fibroblasts were plated (1×10^5^ cells/well) in six-well plates (Falcon/Becton Dickson, Franklin Lakes, NJ) for all experiments and allowed to grow for 48 hours prior to any treatment. Crude cellular protein lysates were prepared using NP-40 lysis buffer supplemented with protease inhibitor, phosphatase inhibitor and 1mM PMSF (Sigma, St. Louis, MO). Total proteins (5 to 10µg) were resolved by 10% SDS-PAGE, electrophoretically transferred to nitrocellulose membranes, and specific proteins were detected by standard Western blotting and chemiluminescence (Western Lightning, Perkin-Elmer, Wellesley, MA). Kodak Molecular Imaging Software (Rochester, NY) was used to perform densitometry on Western blot films and the band intensities were normalized to the loading control. The following primary antibodies were used: phospho-Akt^S473^, total Akt, phospho-PTEN^S380^, total PTEN, phospho-p38 MAPK^T180/Y182^, p38 MAPK, total FAK (Cell Signaling Technologies, Danvers, MA), αSMA (Sigma, St. Louis MO), calponin (DAKO, Carpinteria, CA), GAPDH (Abcam, Cambridge, MA) and phospho-FAK^Y397^ (Invitrogen Corporation Camarillo, CA). The secondary antibodies used were; goat anti-rabbit (sc-2004), goat anti-mouse (sc-2031, Santa Cruz Biotechnology, Inc. Santa Cruz, CA. 95060 U.S.A) and donkey anti-rabbit (NA 934, Amersham/GE Health Care Life Sciences Piscataway, NJ 08855-1327).

### Cell cytotoxicity assay

Cell cytotoxicity was measured by lactate dehydrogenase release assay (LDH5 assay) using an optimized LDH assay kit (Sigma, Cat # DG1340-K). Briefly, fibroblasts were plated in triplicate at a density of 1×10^5^ cells per well in 6 well plates and treated with either CDDO (1µM) or 15d-PGJ2 (5µM) or left untreated for 72 hrs. Release of LDH (nmol/min/mL) was measured at 340 nm using a spectrophotometer as per the manufacturer's protocol. The results were normalized to untreated control samples and plotted as fold change over untreated samples.

### PPRE luciferase reporter assay

Primary lung fibroblasts cultured in 6-well plates were co-transfected using Fugene6 (Roche Applied Science, Indianapolis, IN) with a PPAR-γ-luciferase reporter construct containing three PPREs (a gift from Dr. Brian Seed, Harvard University) [59]. A CMV-β-galactosidase control construct was included as control. After 24 h, the cells were washed and then treated with 15d-PGJ_2_ (5µM) or CDDO (1µM) in medium and harvested after a further 48-hr incubation. Luciferase activity was measured using a luciferase assay system (Promega, Madison, WI) in a luminometer (Packard Instruments, Meriden, CT) and normalized to β-galactosidase activity, determined by a colorimetric assay (Promega). The experiments were carried out in triplicate wells.

### Transfections

Primary HLF cells were plated (5×10^4^cells/well) in 12 well plates (Falcon/Becton Dickson, Franklin Lakes, NJ) and Fugene 6 transfection kit was used as per the manufacturer's protocol (Roche Applied Science, Indianapolis, IN) for transfection. Transfection reactions were carried out using either empty vector pcDNA3.1 or a dominant negative kinase dead Akt (KD-Akt) (a kind gift from Dr. Robert Freeman, University of Rochester, NY USA [34]). Upon transfection, cells were allowed to grow for 16–24 hours, and were then supplemented with 10% FBS for 24 hours followed by the treatment. Cells were lysed using NP-40 lysis buffer and subjected to further analysis as described above. Transfections with HA-FAK (a kind gift from Dr. William Cance, Roswell Park Cancer Institute, Buffalo, NY USA) were performed in a similar manner.

### Indirect immunofluorescence assay

Cells were grown in four well chamber slides and treated as outlined above. Cells were fixed in methanol and stained with an antibody to α-SMA (St. Louis, MO, USA) and with anti-mouse AlexaFluor 488 (Invitrogen Corporation Carlsbad, CA, USA). Slides were mounted with Prolong Gold supplemented with DAPI (Invitrogen Corporation Carlsbad, CA, USA) to visualize the nuclei and analyzed by fluorescence-microscopy using a Zeiss Axio Imager Z.1 Microscope.
